# Supplementary material for: Chemical and Biological Studies of Endophytes Isolated from Marchantia polymorpha
Source: Molecules. 2023 Feb 27;28(5):2202. doi: 10.3390/molecules28052202 (PMC10004590; doi:10.3390/molecules28052202)
Supplement: Supplementary file 1 [file molecules-28-02202-s001.zip › molecules-2226092-supplementary.pdf]

Supplementary Materials

Chemical and Biological Studies of Endophytes Isolated from *Marchantia polymorpha*

Mateusz Stelmasiewicz [1,*], Łukasz Świątek [2] and Agnieszka Ludwiczuk [1,*]

[1] Department of Pharmacognosy with the Medicinal Plant Garden, Medical University of Lublin, Poland
[2] Department of Virology with SARS Laboratory, Medical University of Lublin, Poland
* Correspondence: m.stelmasiewicz7@gmail.com (M.S.); aludwiczuk@pharmacognosy.org (A.L.)

Results

Chromatograms of the fractions obtained from the ethyl acetate extract of the endophytes *Marchantia polymorpha* L.

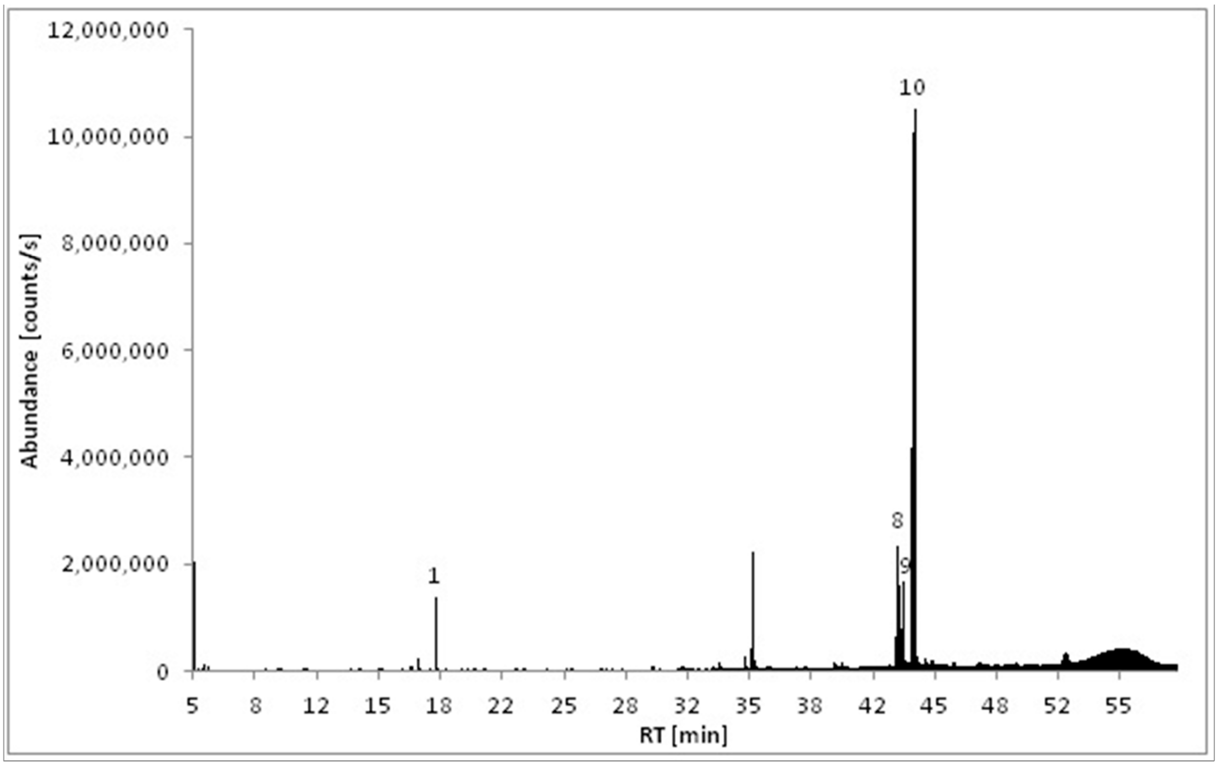

Figure S1.Total ion chromatogram (TIC) of the ethyl acetate extract of *Marchantia polymorpha* endophytes (END2).

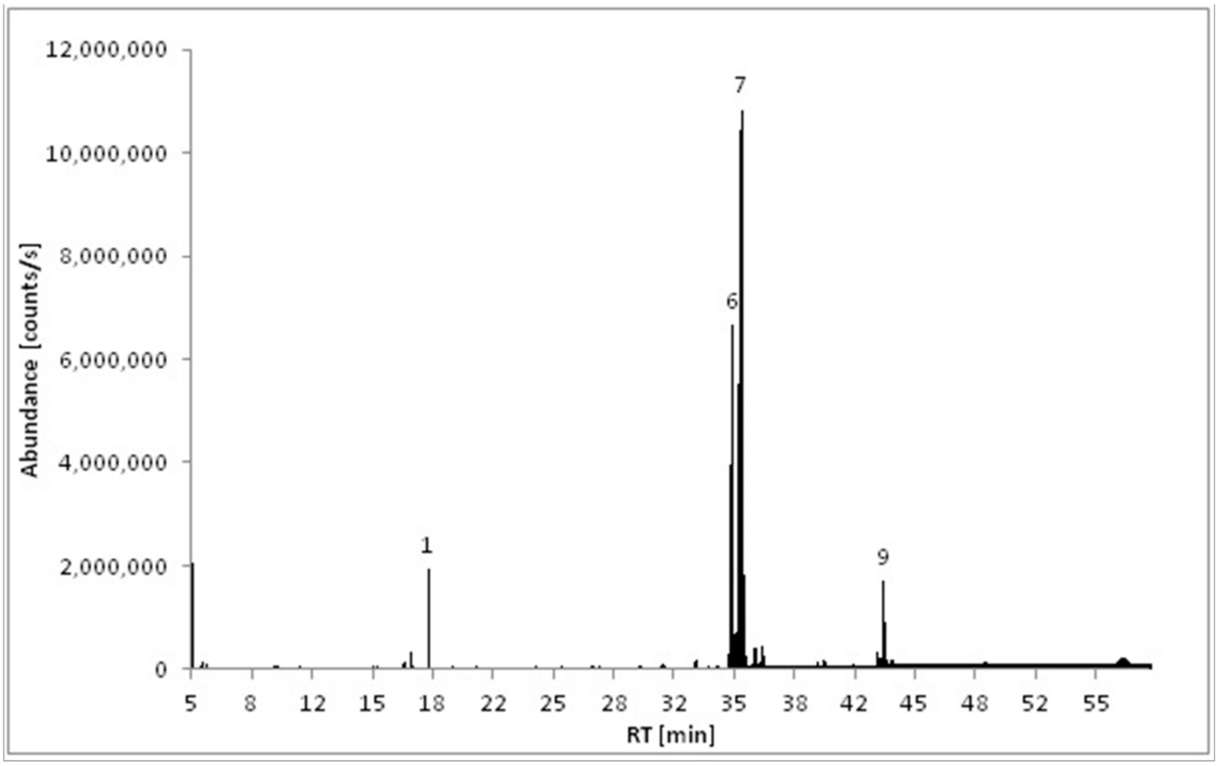

Figure S2. Total ion chromatogram (TIC) of the ethyl acetate extract of *Marchantia polymorpha* endophytes (END3).

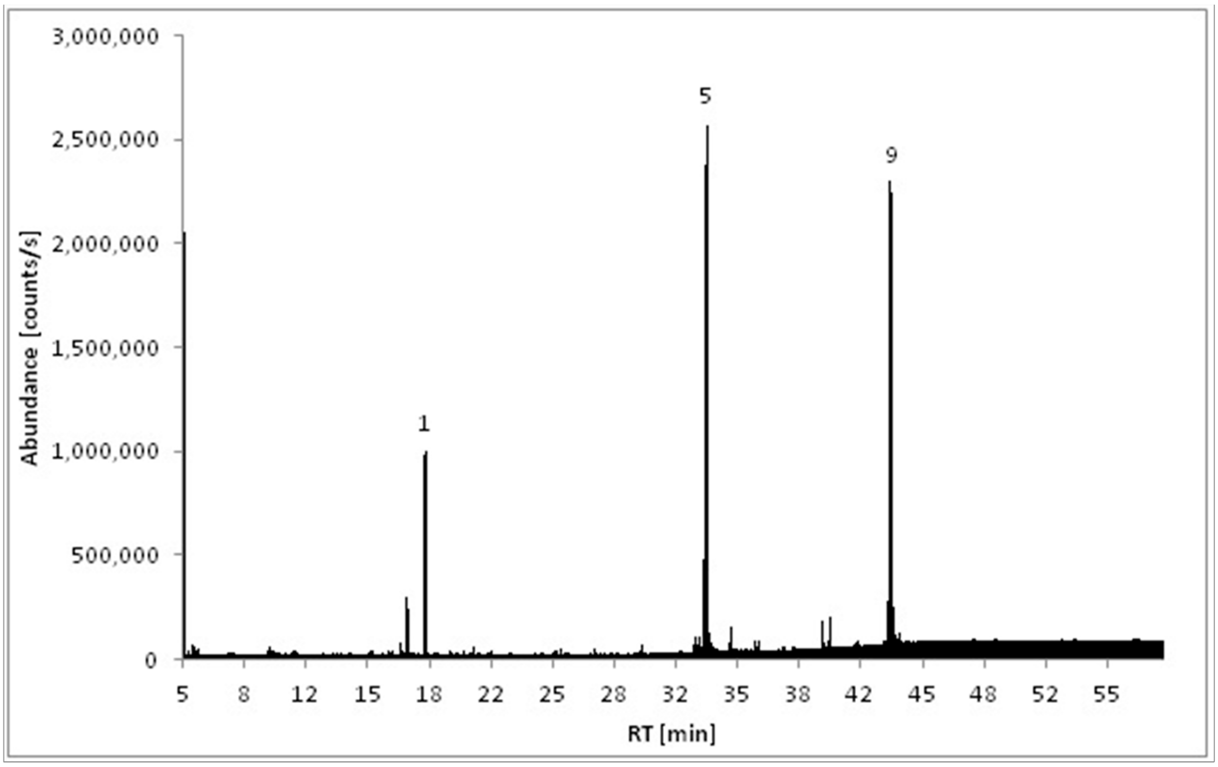

Figure S3. Total ion chromatogram (TIC) of the ethyl acetate extract of *Marchantia polymorpha* endophytes (END4).

Materials and Methods

Cell line maintenance and in vitro experiments

The cytotoxicity of endophyte extracts was evaluated *in vitro* towards normal VERO (ATCC, CCL-81) cells and cancer-derived cell lines – FaDu (ATCC, HTB-43, human hypopharyngeal squamous cell carcinoma), HeLa (human cervical adenocarcinoma, ATCC, CCL-2), and RKO (human colon cancer, ATCC, CRL-2577) using 3-(4,5-dimethylthiazol-2-yl)-2,5-diphenyltetrazolium bromide (MTT) based protocol.

Media used for *in vitro* culturing included Dulbecco Modified Eagle Medium (DMEM, Corning, Tewksbury, MA, USA) used for VERO cells and Modified Eagle Medium (MEM, Corning) used for cancer-derived cell lines. Cell media used in the experiments were supplemented with antibiotics (Penicillin-Streptomycin Solution, Corning) and fetal bovine serum (FBS, Corning) – 10% (cell passaging) and 2% (cell maintenance and experiments). Phosphate buffered saline (PBS) and trypsin were bought from Corning, whereas MTT (3-(4,5-dimethylthiazol-2-yl)-2,5-diphenyltetrazolium bromide) and DMSO (dimethyl sulfoxide) from Sigma (Sigma-Aldrich, St. Louis, MO, USA). Incubation was carried out in a 5% CO_2 atmosphere at 37°C (CO_2 incubator, Panasonic Healthcare Co., Tokyo, Japan).

Stock solutions of extracts were prepared by dissolving the extracts in cell culture grade DMSO (PanReac Applichem). Stock solutions of extracts were stored frozen (-23°C) until used.

The cytotoxicity testing

Cytotoxicity was tested using an MTT-based protocol following a previously described protocol [Świątek, 2021]. Briefly, the cells were passaged into 96-well plates (Falcon, TC-treated, Corning) and, after overnight incubation, treated with serial dilutions of extract stock solutions for 72 h. Afterwards, the media was

removed, cells were washed with PBS, and 10% of MTT solution (5 mg/mL) in cell media was added, and the incubation continued for the next 4 h. Subsequently, the SDS/DMF/PBS (14% SDS, 36% DMF, 50% PBS) solvent was used (100 μL per well) to dissolve the precipitated formazan crystals, and the plates were left at 37°C overnight. Finally, the Synergy H1 Multi-Mode Microplate Reader (BioTek Instruments, Inc. Winooski, Vermont, USA) with Gen5 software (ver. 3.09.07; BioTek Instruments, Inc.) was used to measure the absorbance (540 and 620 nm).

Evaluation of the antiviral properties

The antiviral activity of endophyte extract was tested against HHV-1 (ATCC, Cat. No. VR-260) propagated in the VERO cell line. The antiviral assays involved the influence of extracts on the formation of virus-induced cytopathic effect (CPE), the evaluation of the reduction of infectious titer using the end-point virus titration, and the semi-quantitative assessment of the viral load using Real-Time PCR.

Evaluation of the influence on the virus-induced CPE

The infectious titer of HHV-1 used in this study was 5.5±0.25 log$CCID_{50}$/mL ($CCID_{50}$ – 50% cell culture infectious dose). Briefly, the VERO cells (monolayer) in 48-well plates (Falcon, clear flat bottom TC-treated, Corning) were treated (500 μL/well) with HSV-1 suspension (100-fold $CCID_{50}$/mL) in cell media and incubated for 1 hour, leaving at least two uninfected wells as VERO cell control. Afterwards, the media were removed, monolayers washed with PBS, and the non-toxic concentrations of extracts, the highest concentration not exceeding the CC_{10} values, diluted in cell media were added. The non-infected VERO cells (cell control) and non-treated infected cells (virus control) wells were maintained in media containing 2% FBS. The incubation was conducted until cytopathic effect (CPE) was observed (inverted microscope CKX41, Olympus Corporation, Tokyo, Japan) in virus control, usually approx. 72h. Afterwards, the plates were observed for possible inhibition of CPE by

tested extracts compared to the CPE in virus control, and the results were recorded. Lastly, the plates were thrice frozen (-72°C) and thawed; the samples were collected and stored at -72°C until used in the end-point virus titration assay and DNA isolation.

End-point dilution assay for HHV-1 titration

Samples collected from antiviral assays were subjected to an end-point dilution assay to evaluate the HHV-1 titers. Briefly, the VERO cells (monolayer) in 96-well plates were incubated with ten-fold dilutions of samples (3 replicates) in cell media for 72 hours. Daily observation was conducted to monitor the development of CPE. After the incubation, all media were removed, and the virus infectious titer for each sample was measured using the previously described MTT method. Subsequently, the difference (Δlog) between the endophyte-treated infected cells (END-T) and the virus control (VC) was calculated ($\Delta log = logCCID_{50}VC - logCCID_{50}\ END\text{-}T$). The Δlog values were evaluated for every antiviral assay, and the results were expressed further as means of viral titer reduction. A significant antiviral activity can be reported for extracts decreasing the infectious titer by at least 3 log compared to virus control [Świątek, 2021].

Real-Time PCR for HHV-1 viral load

The DNA isolation was carried out using a commercially available kit (QIAamp DNA Mini Kit, Cat#51304, QIAGEN GmbH, Hilden, Germany) following the manufacturer's instructions. The Real-Time PCR amplification was performed using SybrAdvantage qPCR Premix (Takara Bio Inc., Kusatsu, Shiga Prefecture, Japan) and primers (UL54F – 5′ CGCCAAGAAAATTTCATCGAG 3′, UL54R – 5′ ACATCTTGCACCACGCCAG 3′) on the CFX96 thermal cycler (Bio-Rad Laboratories, Inc., California, USA). The amplification cycle parameters were as follows: initial activation (95°C, 20 secs); cycling (40 repeats: denaturation (95°C, 5

secs), annealing and synthesis (60°C, 30 secs), fluorescence acquisition); melting curve analysis (65-95°C). The HHV-1 viral load in the tested samples was assessed in relation to virus control based on the relative quantity (ΔCq) method using CFX Manager™ Dx Software (Bio-Rad Laboratories).

Supplementary material references

Świątek, Ł.; Sieniawska, E.; Sinan, K.I.; Maciejewska-Turska, M.; Boguszewska, A.; Polz-Dacewicz, M.; Senkardes, I.; Guler, G.O.; Bibi Sadeer, N.; Mahomoodally, M.F.; Zengin, G. LC-ESI-QTOF-MS/MS Analysis, Cytotoxic, Antiviral, Antioxidant, and Enzyme Inhibitory Properties of Four Extracts of *Geranium pyrenaicum* Burm. f.: A Good Gift from the Natural Treasure. *Int. J. Mol. Sci.* 2021, *22*, 7621. https://doi.org/10.3390/ijms22147621
